# Effect of parecoxib on postoperative delirium in patients with hyperlipidemia: a randomized, double-blind, single-center, superiority trial

**DOI:** 10.1097/JS9.0000000000002286

**Published:** 2025-02-04

**Authors:** Zeng Daojun, Tang Yuling, Xu Yingzhe, Ana Kowark, Mark Coburn, Zhao Yue, Zhang Qixin, Zhang Daiying, Peng Tao, Duan Xiaoxia

**Affiliations:** a Department of Anaesthesiology, The Affiliated Hospital, Southwest Medical University, Luzhou, Sichuan Province, China; b Anaesthesiology and Critical Care Medicine Key Laboratory of Luzhou, Southwest Medical University, Luzhou, Sichuan Province, China; c Operating room, The Affiliated Hospital, Southwest Medical University, Luzhou, Sichuan Province, China; d Department of Anaesthesiology and Intensive Care Medicine, University Hospital Bonn, Venusberg-Campus 1, Bonn, Germany

**Keywords:** hyperlipidaemia, inflammation, parecoxib, post operative delirium

## Abstract

**Background:**

Hyperlipidemia has been implicated in the higher risk of developing postoperative delirium. Prostaglandin endoperoxide synthase-2 mediates neuroinflammatory processes in postoperative delirium. This study aims to investigate whether preoperative administration of parecoxib is more efficient than a placebo in averting postoperative delirium in patients with hyperlipidemia.

**Materials and methods:**

In this randomized, double-blind, superiority trial, participants with hyperlipidemia were randomized in a 1:1 ratio to receive parecoxib (40 mg parecoxib administered intravenously before anesthesia induction) or placebo (normal saline). The primary outcome was postoperative delirium incidence within three days, with a 5.4% difference set as the superiority threshold. Secondary outcomes were cumulative incidences of emergence delirium and prostaglandin endoperoxide synthase-2 levels, inflammatory cell counts, and pain score on postoperative day 1 and postoperative adverse events.

**Results:**

This trial conducted between August 2023 and August 2024 at a tertiary hospital in China included 452 adults with hyperlipidemia, with 226 in the parecoxib group and 226 in the placebo group. The incidence of postoperative delirium in the parecoxib group was 13.72%, a reduction of 12.39% compared to the placebo group (hazard ratio, 0.491; 95% confidence interval: 0.318 to 0.759; *P* < 0.001). The parecoxib group also had a lower incidence of emergence delirium, prostaglandin endoperoxide synthase-2 levels, white blood cell counts and neutrophil, and pain scores on postoperative day 1. The occurrence of adverse events was comparable between the two groups. Prostaglandin endoperoxide synthase-2 expression levels, white blood cell counts, and pain scores mediated the reduction of postoperative delirium incidence by parecoxib.

**Conclusion:**

Parecoxib may help in reducing the hyperlipidemia-related postoperative delirium incidence. The effective anti-inflammatory activity of prostaglandin endoperoxide synthase-2 inhibition by parecoxib and postoperative pain control may be important mechanisms for preventing postoperative delirium.

## Introduction

Postoperative delirium (POD) manifests as sudden cerebral dysfunction, changes in cognitive function, and disorientation, which are rapidly developing and frequently fluctuating within a short period^[1]^. The combined occurrence of POD among elderly Chinese individuals amounted to 18.6%^[2]^. In addition to causing short-term cognitive impairment, POD can also lead to permanent cognitive decline and dementia^[3]^, significantly impairing patients’ activities of daily living and increasing the incidence of death^[4]^. Numerous cohort studies, conducted on a large scale, have revealed an association between elevated lipoprotein levels and the increased likelihood of developing POD^[5,6]^. A preliminary investigation has indicated that individuals with hyperlipidemia are more likely to experience an elevated risk, as well as a prolonged period, of POD^[7]^. Therefore, hyperlipidemia constitutes a significant risk factor for the development of POD.

Studies have consistently confirmed that hyperlipidaemia can activate glial cells^[8]^, induce neuroinflammation^[9]^, and accelerate neurodegenerative changes and cognitive decline^[10]^. Perioperative injury can exacerbate inflammatory reactions^[11]^, playing a part in the development of numerous perioperative neurological disorders, including POD^[12]^. Prostaglandin endoperoxide synthase-2 (PTGS2/COX-2) is a key regulator of inflammatory mediators^[13]^ and promotes the occurrence of neuroinflammation^[14]^. A previous study confirmed that increased PTGS2 is directly related to postoperative cognitive impairment^[15]^. Inhibition of PTGS2 can cause a reduction in levels of central^[16]^ and peripheral inflammatory factors, which is associated with improved postoperative cognitive function^[17]^. Therefore, inhibiting PTGS2 may reduce POD emergence in patients with hyperlipidemia.

Parecoxib is a selective PTGS2 inhibitor, extensively utilized in perioperative analgesia. Studies have indicated that parecoxib can mitigate the POD risk in elderly surgical patients, accompanied by a decrease in inflammatory levels and pain intensity^[18,19]^. However, it remains unclear whether parecoxib influences the prevalence of POD in individuals with hyperlipidemia. Consequently, this particular study aimed to investigate the effects of administering parecoxib preoperatively on POD occurrence in patients with hyperlipidemia.

## Material and methods

### Selection of study participants

This randomized, double-blind, single-center, superiority trial was conducted at at a tertiary hospital in China between August 2023 and August 2024 and was granted approval from the hospital’s ethics committee on 19 June 2023 (ethical approval number XXX). From all individuals who participated in the study, written confirmation of informed consent was duly procured. The trial was registered in the Chinese clinical trial registry (registration number XXX) on 24 July 2023. This study complied with the Consolidated Standards of Reporting Trials reporting guideline^[20]^ and the Declaration of Helsinki. The trial protocol details are accessible in Supplementary Digital Content, Text 1, available at: http://links.lww.com/JS9/D853.

Hyperlipidemia is highly prevalent among gastrointestinal surgical patients^[21]^, and POD is also a common complication in this population^[22]^. Therefore, this study targeted patients who were undergoing a radical resection surgery for the treatment of colorectal cancer. The criteria for participant inclusion were as follows:

1) Presence of hyperlipidemia preoperatively; 2) age ≥ 18 years, ≤ 100 years; 3) American Society of Anaesthesiologists (ASA) physical status classification I–III. According to the latest edition of the Chinese guideline for lipid management (2023), a slight elevation (triglycerides ≥ 1.7 mmol · L^−1^, total cholesterol ≥ 5.2 mmol · L^−1^, and high-density lipoproteins ≤ 1.0 mmol · L^−1^, at least one of the above must be satisfied) of blood lipids in individuals at low risk for atherosclerotic cardiovascular disease was considered hyperlipidemia^[23]^.

Exclusion criteria included 1) preoperative pre-existing delirium state; 2) history of head trauma, psychiatric, or neurological disorders; 3) history of antibiotic abuse; 4) Child-Pugh score for liver function ≥ 6 points or creatinine clearance rate < 30 mL · min^−1^; 5) allergic to or use of long-term non-steroidal anti-inflammatory drug (NSAID); 6) history of serious adverse drug reactions; 7) patients with a history of gastrointestinal bleeding or perforation following NSAID use; 8) patients with active peptic ulcer or gastrointestinal bleeding, inflammatory bowel disease; 9) patients who are in the third trimester of pregnancy or breastfeeding; 10) pre-existing severe cardiovascular and cerebrovascular diseases; and 11) history of use of lipid-lowering drugs within 2 weeks prior to surgery.

### Randomization and blinding

Non-blinded researchers were responsible for concealing the allocation and performing randomization for all study participants. A random assignment (1:1) was used to allocate patients to either the parecoxib or placebo group using a computer randomization system. Parecoxib 40 mg (Chengdu Baiyu Pharmaceutical Co., Ltd., State medical permission number: H20203212) was dissolved in 5 mL of normal saline and packaged in a 5 mL sterile syringe^[24]^. The parecoxib group received an intravenous injection of this packaged solution 15 min before anesthesia induction, while the placebo group received an injection of an identical volume of saline simultaneously. All medications were enclosed in indistinguishable, opaque envelopes and distributed to the respective anesthesiologists by a research assistant. The physicians, anesthesiologists, patients, and other team members remained blinded to the study group allocations and interventions for the study’s full duration.

### Anesthetic surgical strategy

The induction of anesthesia and endotracheal intubation were facilitated by intravenous administration of penehyclidine hydrochloride (0.01 mg · kg^−1^), sufentanil (0.3 to 0.5 µg · kg^−1^), propofol (1.5 to 2.5 mg · kg^−1^), and rocuronium bromide (0.6 mg · kg^−1^). Subsequently, participants were mechanically ventilated with the following respiratory parameters: a respiratory rate of 12–18 breaths per minute, a tidal volume of 6–8 mL · kg^−1^, an inspiratory to expiratory ratio of 1:2, and inspired oxygen at 50%, all adjusted to a stabilising end-tidal carbon dioxide level of 35–45 mmHg. To maintain anesthesia, remifentanil (0.08–0.20 μg · kg^−1^ · min^−1^), cisatracurium besylate (1–3 μg · kg^−1^ · min^−1^), and sevoflurane (1–2.5% concentration) were employed to keep the bispectral index (BIS) between 40 and 60. At the end of the surgical procedure, initial recovery for the patient occurred in the operating room, which was followed by a transition to the post-anesthesia care unit (PACU) for comprehensive recovery procedures. Patients returned to the ward after meeting pre-established criteria (Aldrete score ≥ 9)^[25]^. For postoperative pain control, sufentanil 0.04 μg · kg^−1^ · h^−1^, butorphanol tartrate 1.5 μg · kg^−1^ · h^−1^, and granisetron 0.18 mg · kg^−1^ were mixed in 200 mL normal saline and administered through a disposable intravenous infusion pump for patient-controlled analgesia.

In this study, the participants underwent various surgical interventions, including transverse colectomy, sigmoid colectomy, right hemicolectomy, left hemicolectomy, Dixon’s procedure, Hartmann’s procedure, and Miles’ procedure. The general protocol is as follows: After the induction of anesthesia, patients are positioned in the supine position with the head slightly lower. Four to five surgical ports are created in the umbilical, upper abdominal, and lower abdominal regions. Upon entering the abdominal cavity, a thorough exploration of the organs and mesenteries is conducted to pinpoint the location of the lesion. Subsequently, the tumor, along with the affected mesentery and lymph nodes, is excised. The resected specimen is extracted through a midline incision at the umbilicus. Following this, an end-to-end intestinal anastomosis is performed. For patients undergoing Hartmann’s or Miles’ procedure indicated for rectal cancer, a proximal colostomy is created, with the distal bowel either being closed or removed.

### Data acquisition and outcome assessment

#### Clinical data collection

Data pertaining to the patients were collected from electronic medical records (iMedical HIS 9.0.1, Donghua Standard Edition Digital Hospital), encompassing basic details such as age, sex, body mass index (BMI), and educational background. Medical details, including history of stroke, carotid artery stenosis, coronary heart disease, hypertension, valvular disease, and diabetes, were also collected. Smoking status and alcohol intake were obtained from the records. Additionally, blood lipid test results at admission, PTGS2 levels on the day before the surgery, and serum levels of inflammatory cells (lymphocyte, neutrophil, and white blood cell counts) before surgery were collected. Perioperative data, including ASA classifications, duration of operation, intraoperative blood pressure variation, blood loss, and presence of hypothermia, were documented. Using this comprehensive dataset, the age-adjusted Charlson Comorbidity Index (ACCI) score was calculated to assess the comorbidity burden in patients^[26]^.

#### Primary outcome assessment

In this study, the principal outcome measured was the POD within a 3-day period post-surgery. To rule out preoperative delirium and evaluate POD, the assessment of patients in this study was conducted utilizing the 3-minute diagnostic interview for the Confusion Assessment Method (3D-CAM) scale, which was administered between 4:00 PM and 8:00 PM on each of the first three days following surgery^[27]^. All 3D-CAM assessments were conducted by researchers who had undergone specialized training by neurologists. Beyond this time frame, POD was screened by examining medical records and consultation notes.

For patients who developed POD, severity and occurrence days of POD were recorded. Severity of delirium was assessed using the method developed by Vasunilashorn and colleagues, which awards a score between zero and 20 based on the positive results from the 3D-CAM scale. Higher scores implied more severe delirium, and this evaluation method is characterized by its high sensitivity and specificity^[28]^.

#### Secondary outcome assessment

Secondary outcomes included the cumulative incidence of emergence delirium (ED); levels of PTGS2, inflammatory cells (lymphocyte, neutrophil, and white blood cell counts), and the pain score using the visual analog scale (VAS) on postoperative day 1; and postoperative adverse events. ED was assessed using the Riker Sedation-Agitation scale, which was conducted every 15 min during the stay in the PACU and until discharge. A score of < 4 or > 4 at any time point was indicative of ED^[29]^. In accordance with the Common Terminology Criteria for Adverse Events 5.0 definition, adverse events occurring during the postoperative hospital stay were assessed, with mortality and cognitive impairment followed up to 3 months postoperatively^[30]^. The Telephone Interview for Cognitive Status-Modified (TICS-M) served as the standard for the evaluation of cognitive dysfunction^[31]^.

Blood samples were collected from patients on the day preceding the surgery and in the morning of the first postoperative day for the determination of PTGS2 levels. At the time of collection, patients were in a supine position and resting state. EDTA anticoagulant tubes were employed to gather 5 mL of venous blood from the patients’ elbows, which were temporarily refrigerated at 2–8°C. Subsequently, the samples were centrifuged at a rate of 3,000 revolutions per minute for 15 minutes and stored at a temperature of −80°C until analysis. To quantify the expression level of PTGS2 in the blood samples, an ELSIA kit specific for human PTGS2 (YMS11256-A; Chengdu Yuannuo Tiancheng Technology Co., Ltd., China) was utilized.

### Statistical analysis

Drawing from a previous study^[7]^, it was projected that among the patients with hyperlipidemia in the placebo group, the incidence of POD was 21.6%. We set the superiority threshold as quarter of this incidence, i.e., 5.4%, and expected that parecoxib would reduce the incidence of POD in patients with hyperlipidemia by 15%^[18]^. Therefore, we set a one-tailed α = 0.05 with 90% power (1–β) and the sample size ratio of the two groups to 1:1. The calculated number of patients per group was 215. Taking into account a possible dropout rate of 5%, we included a total of 452 patients, with an equal distribution of 226 patients in each group. The sample size was calculated using PASS 15.05 software.

Data were analyzed using modified intention-to-treat. Normality was assessed using the Kolmogorov–Smirnov test; normally distributed data (mean ± standard deviation) were compared using Student’s *t*-test, while non-normally distributed data (median [IQR]) were compared using the Mann–Whitney *U* test. Categorical data (rates/proportions) were analyzed using χ^2^ or Fisher’s exact tests. Kaplan–Meier curves and the one-sided log-rank test assessed time to delirium. Subgroup analyses and forest plots were generated to further explore the outcomes of delirium. To analyze the POD severity scores collected continuously over three days, inter-group differences were assessed using generalized estimating equations. For other outcome indicators, a one-sided Student *t*-test, a Mann–Whitney *U* test, or a one-sided proportion test were used to compare differences between groups. Comparisons of other outcomes used one-sided *t*-tests, Mann–Whitney *U* tests, or proportion tests. Mediation analysis explored the roles of inflammation and pain in the incidence of POD and parecoxib. Restricted cubic spline regression (RCS, 4 nodes) analyzed inflammatory markers’ relationship with POD risk. Statistical analyses were conducted using SPSS 25.0 and R 4.3.2, with *P* < 0.05 indicating significance.

## Results

### Patient demographics

A total of 678 patients were recruited for this study. Following the exclusion of 226 patients, 452 patients were randomly assigned to two groups. A total of 18 patients (3.98%) were removed from the analysis due to intensive care unit (ICU) admission and reoperation. Ultimately, a total of 434 patients completed the study, comprising 219 and 215 patients in the parecoxib and placebo groups, respectively (SDC, Figure 1. http://links.lww.com/JS9/D805).

We observed no significant differences in the preoperative baseline characteristics (age, sex, BMI, educational background, lipid profiles, PTGS2 levels, inflammatory cell counts, medical history, ACCI scores, and ASA classifications) between groups. Moreover, no significant differences were discovered in the intraoperative conditions, such as estimated blood loss, surgical duration, blood pressure variability, and presence of hypothermia (Table 1).

**Table 1:** Preoperative and intraoperative clinical characteristics of study participants

Variable	Overall	Parecoxib	Placebo	*P*-value
		*n* = 226	*n* = 226	
**Preoperative**				
Age, median (IQR), year	65 (15)	64 (15)	65 (15)	0.285
Male, *N* (%)	262 (57.96%)	130(57.52%)	132 (58.41%)	0.849
BMI, (mean ± SD), kg · m^−2^	22.62 ± 3.26	22.78 ± 3.34	22.45 ± 3.19	0.272
Educational background (%)				
No formal education and primary school	245 (54.23%)	120 (53.10%)	125 (55.31%)	0.588
Junior or senior high school	166 (36.73%)	84 (37.17%)	82 (36.28%)	
Bachelor or higher	41 (9.07%)	22 (9.73%)	19 (8.41%)	
TG, median (IQR), mmol/L	1.37 (0.86)	1.33 (0.81)	1.50 (0.90)	0.225
TC, median (IQR), mmol/L	5.18(1.36)	5.19 (1.42)	5.14 (1.37)	0.673
LDL-C, median (IQR), mmol/L	3.12 (0.97)	3.17 (0.93)	3.10 (1.02)	0.613
HDL-C, median (IQR)), mmol/L	1.15 (0.50)	1.15 (0.49)	1.16(0.49)	0.623
PTGS2, median (IQR), ng/mL	36.92 (9.14)	36.92 (9.91)	36.92 (8.52)	0.400
Lymphocyte, median (IQR), 10^9^ L^−1^	1.43 (0.77)	1.43 (0.77)	1.42 (0.76)	0.917
Neutrophil, median (IQR), 10^9^ L^−1^	3.70 (1.90)	3.82 (1.92)	3.56(1.91)	0.605
White blood cell, median (IQR), 10^9^ L^−1^	5.89 (2.17)	5.96 (2.26)	5.88 (2.04)	0.815
Stroke (%)	28 (6.19%)	15 (6.64%)	13 (5.75%)	0.696
Carotid artery stenosis, *N* (%)	54 (11.95%)	28 (12.39%)	26 (11.50%)	0.772
Coronary heart disease, *N* (%)	34 (7.52%)	16 (7.08%)	18 (7.96%)	0.721
Hypertension, *N* (%)	130 (28.76%)	58 (25.66%)	72 (31.86%)	0.146
Valvular disease, *N* (%)	26 (5.75%)	17 (7.52%)	9 (3.98%)	0.106
Diabetes mellitus, *N* (%)	63 (13.94%)	27 (11.95%)	36 (15.93%)	0.222
Smoking, *N* (%)	134 (29.65%)	70 (30.97%)	64 (28.32%)	0.516
Alcoholism, *N* (%)	97 (21.46%)	47 (20.80%)	50 (22.12%)	0.731
ACCI, median (IQR), score	5 (3)	5 (3)	5 (3)	0.868
ASA (≥ III), *N* (%)	140 (30.97%)	63 (27.88%)	77 (34.07%)	0.154
**Intraoperative**				
Surgery duration, median (IQR), min	240 (85)	240 (86)	240 (80)	0.838
Estimated blood loss, median (IQR), ml	50 (50)	50 (50)	50 (50)	0.564
IBPV^a^, median (IQR)	0 (2)	0 (1)	0 (1)	0.177
Hypothermia^b^, *N* (%)	151 (33.41%)	70 (30.97%)	81 (35.84%)	0.273

ACCI, age-adjusted Charlson comorbidity index; ASA, American Society of Anesthesiologists; BMI, body mass index; HDL-C, high-density lipoprotein cholesterol; IBPV, intraoperative blood pressure variability; IQR, interquartile range; LDL-C, low-density lipoprotein cholesterol; PTGS2, prostaglandin-endoperoxide synthase 2; SD, standard deviation; TC, total cholesterol; TG, triglycerides.

^a^ Total number of episodes with mean arterial pressure fluctuations exceeding 20% of baseline values.

^b^ Nasopharyngeal temperature < 36°C.

### POD according to groups

**Figure 1.**
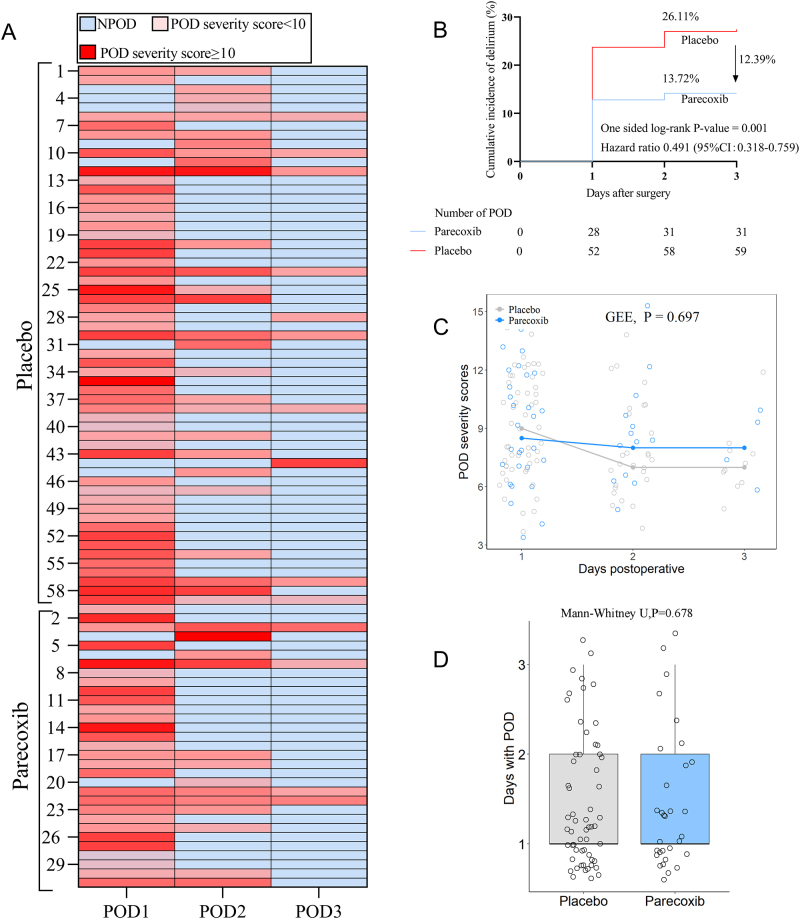
Comparison of POD occurrence between the two groups. (**A**) POD onset, days, and severity; (**B**) cumulative incidence of POD by treatment, the one-sided log-rank test was used to compare delirium distribution; (**C**) POD severity scores between the parecoxib and placebo groups; (**D**) days with POD between the parecoxib and placebo groups. In the scatter plot of POD severity scores on postoperative day 3, trend lines were drawn connecting the median values for each group. The blue dots represent the parecoxib group, while the grey dots represent the placebo group. In the box and whisker plot, the box spans the IQR, bounded by the 25th percentile and the 75th percentile. The horizontal line within the box indicates the median of the data. The upper whisker extends from the 75th percentile to the maximum value, while the lower whisker extends from the 25th percentile to the minimum value. The differences in the distribution of POD occurrence days between the groups were compared using the Mann–Whitney *U* test.

IQR, interquartile range; POD, postoperative delirium; POD1, postoperative day 1; POD2, postoperative day 2; POD3, postoperative day 3; GEE, generalized estimating equations.

The onset, days, and severity of POD within the first 3 days postoperatively are shown in Fig. 1A. The overall POD incidence on postoperative days 1, 2, and 3 were 17.70%, 8.85%, and 3.10%, respectively, with a total of 90 patients (19.91%) experiencing POD. Compared with the placebo group, it was found that the POD incidence was significantly lower in the parecoxib group on postoperative day 1 (12.39% vs. 22.57%, *P* = 0.003) and 2 (5.75% vs. 12.00%, *P* = 0.015), but not on postoperative day 3 (1.79% vs. 4.48%, *P* = 0.086). As shown in Fig. 1B, the cumulative POD incidence was 13.72% in the parecoxib group, which was 12.39% lower than that in the placebo group (26.11%), exceeding the 5.4% efficacy threshold, indicating significant superiority (hazard ratio [HR], 0.491, 95% confidence interval [CI]: 0.318 to 0.759; *P* = 0.001). Among patients who developed POD, there were no significant differences in the POD severity scores (Fig. 1C), and days with POD (Fig. 1D) between the two groups (SDC, Table 1. http://links.lww.com/JS9/D802).

## Subgroup analysis of POD incidence

**Figure 2.**
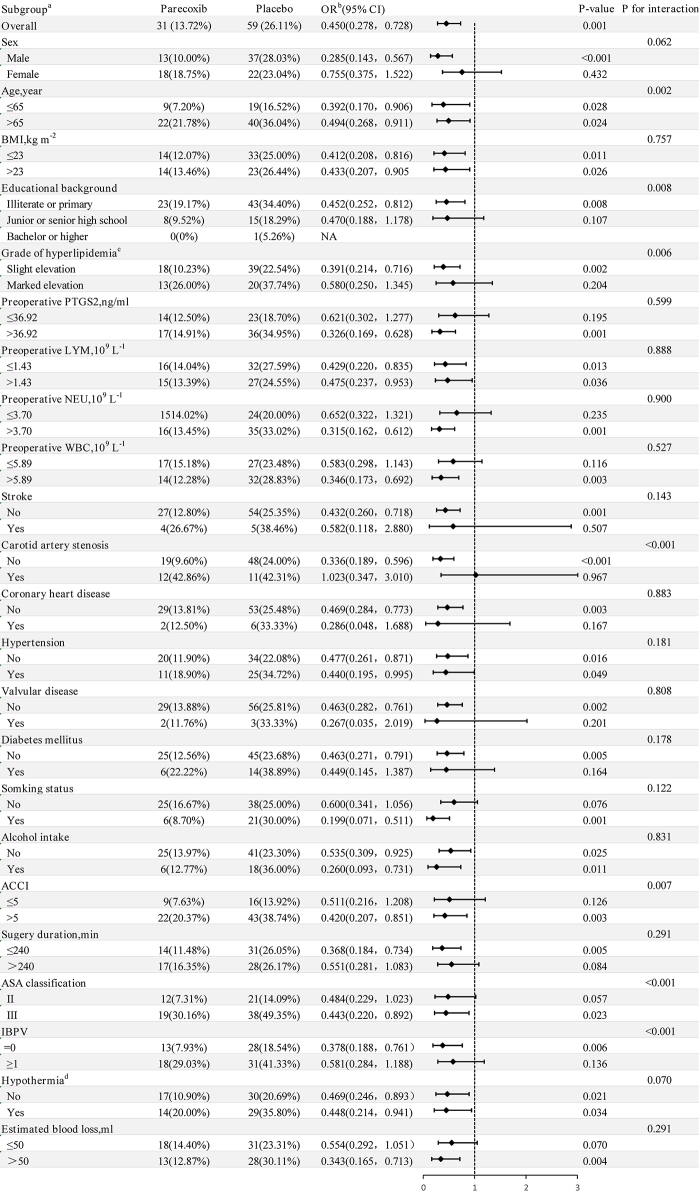
Subgroup analysis of POD incidence.

^a^Subgroups were divided using a predefined method, dichotomising quantitative data at the mean or median. ^b^Odds ratios (ORs) were determined via univariate logistic regression. ^c^According to the Chinese guidelines for lipid management (2023), triglycerides (TG) ≥ 2.3 mmol · L^−1^, total cholesterol (TC) ≥ 6.2 mmol · L^−1^, low-density lipoprotein cholesterol (LDL-C) ≥ 4.1 mmol · L^−1^, or non-high-density lipoprotein cholesterol (non-HDL-C) ≥ 4.9 mmol · L^−1^ are used as the boundary to classify hyperlipidemia into slight and marked elevation.NA, no analysis was conducted due to insufficient data; BMI, body mass index; PTGS2, prostaglandin-endoperoxide synthase 2; LYM, lymphocyte counts; NEU, neutrophil counts; WBC, white blood cell; ACCI, age-adjusted Charlson comorbidity index; ASA, American Society of Anaesthesiologists; IBPV, intraoperative blood pressure variability.

A predefined subgroup analysis identified a subset of patients in whom the effect of parecoxib on POD incidence was most pronounced. These patients either had no formal education or attended only primary school, a slight elevation in lipid levels, no carotid artery stenosis, an ASA score of III, ACCI scores ≥ 5, and no episode of intraoperative blood pressure variability (*P* for interaction < 0.05). In contrast, the following subgroups did not interact significantly with parecoxib: sex, preoperative PTGS2 levels, preoperative neutrophil counts, preoperative lymphocyte counts, preoperative white blood cell counts, BMI, stroke, coronary artery disease, hypertension, diabetes, valvular disease, smoking status, and alcohol intake, as well as surgery duration, intraoperative hypothermia, and estimated blood loss. Notably, the age subgroups interacted significantly with parecoxib (*P* for interaction < 0.05); however, the significance of the odds ratio (OR) values remained unaltered (Fig. 2).

### Secondary outcome between groups

**Figure 3.**
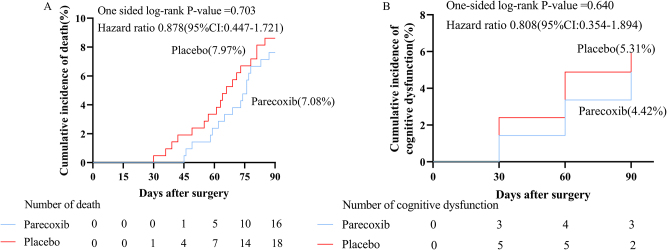
Survival analysis of 3-month postoperative mortality and cognitive dysfunction.

In comparison to the placebo group, ED in the PACU occurred less frequently (15.04% vs. 25.66%, HR, 0.551; 95% CI: 0.361 to 0.841; *P* = 0.005, SDC, Figure 2A. http://links.lww.com/JS9/D806) in the parecoxib group. Significant differences were observed in the level of PTGS2 (32.24 [11.16] vs. 43.05 [9.81] ng · Ml^−1^, *P* < 0.001, SDC, Figure 2E. http://links.lww.com/JS9/D806), neutrophil counts (7.82 [2.73] vs. 8.59 [3.11] × 10^9^ L^−1^, *P* < 0.001, SDC, Figure 2D. http://links.lww.com/JS9/D806), white blood cell counts (9.43 [2.82] vs. 10.20 [3.04] × 10^9^ L^−1^, *P* < 0.001, SDC, Figure 2 F. http://links.lww.com/JS9/D806), and the VAS pain score on postoperative day 1 (3 [1] vs. 3 [1], *P* < 0.001, SDC, Figure 2B. http://links.lww.com/JS9/D806). There were no significant differences between the two groups in terms of postoperative lymphocyte counts on day 1 and postoperative adverse events, including 3-month mortality (7.08% vs. 7.97%, HR, 0.878; 95% CI: 0.447 to 1.721, *P* = 0.703, Fig. 3A) and cognitive impairment (4.42% vs. 5.31%, HR, 0.808; 95% CI: 0.354 to 1.894, *P* = 0.640, Fig. 3B) (SDC, Table 2. http://links.lww.com/JS9/D803).

The one-sided log-rank test was used to compare delirium distribution. **(A)** Cumulative incidence of mortality at 3 months post-surgery according to treatment; **(B)** cumulative incidence of cognitive dysfunction at 3 months post-surgery according to treatment.

### Mediation analysis

**Figure 4.**
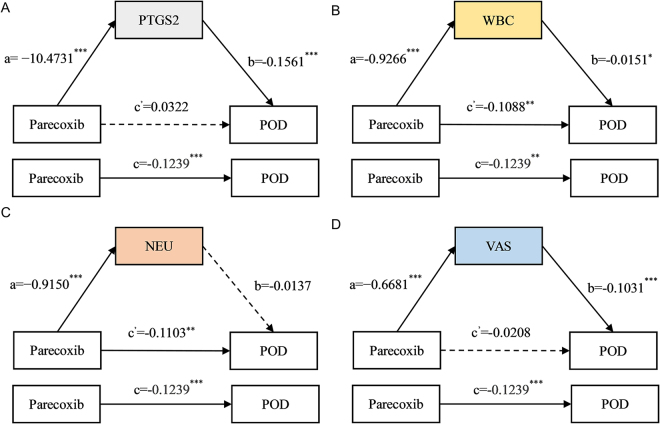
Mediation analysis of **(A)** PTGS2, **(B)** WBC, **(C)** NEU, and **(D)** VAS on the first postoperative day in the incidence of POD between parecoxib and POD.

POD, postoperative delirium; PTGS2, prostaglandin-endoperoxide synthase 2; WBC, white blood cell; NEU, neutrophil; VAS, visual analog scale; ^*^P < 0.05, ^**^P < 0.01; ^***^P < 0.001.

To further investigate whether parecoxib indirectly affects POD by alleviating postoperative inflammation and pain, we conducted a mediation analysis. We used postoperative day 1 PTGS2 levels, neutrophil counts, white blood cell counts, and VAS scores separately as a “mediator,” parecoxib as the independent variable, and POD incidence as the outcome variable. The results showed that PTGS2 (c’ = 0.0322, 95% CI: − 0.0462 to 0.1100, *P* = 0.400, b = − 0.1561, 95% CI: − 0.2160 to −0.1100, *P* <0.001, Fig. 4A) and VAS scores (c’ = − 0.0208, 95% CI: − 0.0959 to 0.0600, *P* = 0.590, b = − 0.1031, 95% CI: − 0.1497 to −0.0700, *P* <0.001, Fig. 4D) exhibited complete mediating effects, white blood cell counts (c’ = − 0.1088, 95% CI: − 0.1790 to −0.0400, *P* = 0.003, b = − 0.0151, 95% CI: − 0.0357to −0.0000, *P* = 0.022, Fig. 4B) exhibited partial mediating effects, whereas neutrophil counts (c’ = − 0.1103, 95% CI: − 0.1805 to −0.0300, *P* = 0.004, b = − 0.0137, 95% CI: − 0.0343 to −0.0000, *P* = 0.072, Fig. 4C) did not demonstrate mediating effects (SDC, Table 3. http://links.lww.com/JS9/D804).

### Associations between PTGS2, neutrophil counts, white blood cell counts, and the risk of POD

RCS analysis was employed to evaluate the association between the PTGS2 levels, as well as white blood cell counts and neutrophil counts on the first postoperative day, and the risk of POD.

**Figure 5.**
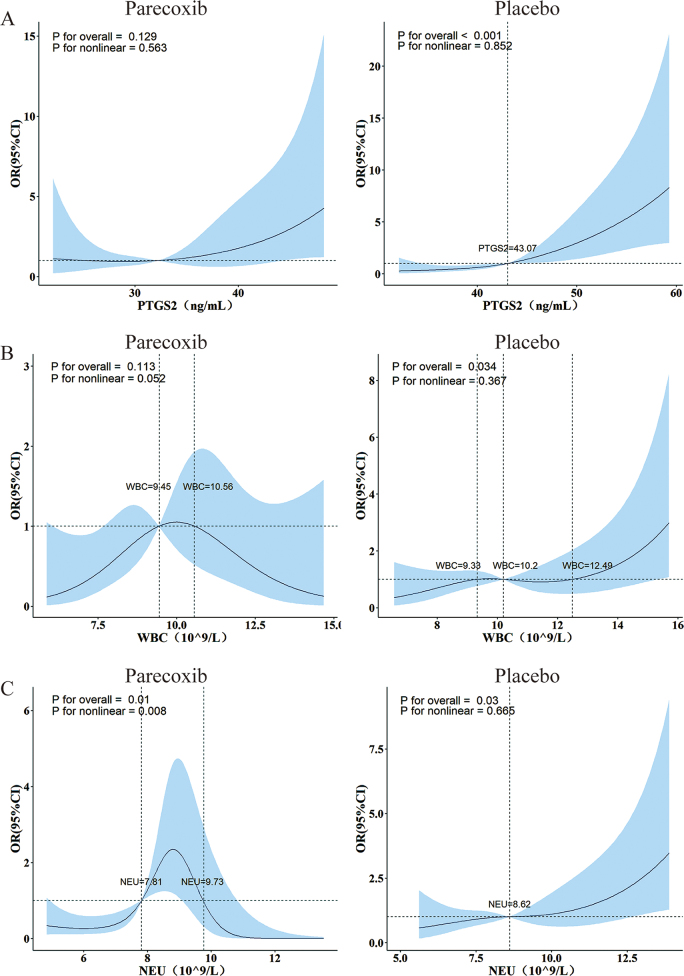
Dynamic risk relationship between **(A)** PTGS2, **(B)** NEU, and **(C)** WBC on the first postoperative day and POD.

It revealed that PTGS2 levels, white blood cell counts, and neutrophil counts were all positively correlated with POD risk in a linear manner in the placebo group. The OR values exceed 1 when PTGS2 levels > 43.07 ng · mL^−1^ (*P* for overall < 0.001, *P* for nonlinear = 0.852, Fig. 5A), white blood cell counts > 12.49 × 10^9^ L^−1^ (*P* for overall = 0.034, *P* for nonlinear = 0.367, Fig. 5B), and neutrophil counts > 8.62 × 10^9^ L^−1^ (*P* for overall = 0.030, *P* for nonlinear = 0.665, Figure 5C), respectively.

The OR values were calculated by adjusting the educational background, hyperlipidemia grade, carotid artery stenosis, ACCI scores, ASA classifications, and the number of blood pressure variations by RCS. The solid black line represents the estimated OR value, and the blue band represents its 95% confidence interval.ACCI, age-adjusted Charlson comorbidity index; ASA, American Society of Anaesthesiologists; OR, odds ratio; POD, postoperative delirium; PTGS2, prostaglandin-endoperoxide synthase 2; NEU, neutrophil; WBC, white blood cell.

In the parecoxib group, PTGS2 levels (*P* for overall = 0.113, *P* for nonlinear = 0.052, Figure 5A) and white blood cell counts (*P* for overall = 0.081, *P* for nonlinear = 0.052, Figure 5B) showed no correlation with the risk of POD. Neutrophil counts exhibited a non-linear, volcanic-shaped relationship with POD risk. The OR values exceed 1 when neutrophil counts were between 7.81 and 9.73 × 10^9^ L^−1^ (*P* for overall = 0.010, *P* for nonlinear = 0.008, Figure 5C).

## Discussion

According to this study, we disclosed that the incidence of POD in patients who received preoperative parecoxib was reduced significantly, demonstrating great superiority compared to the placebo group. Among patients who experienced POD, no significant difference was detected between both groups regarding the severity and occurrence days of POD.

POD, defined as a reversible syndrome of cognitive dysfunction, is a prevalent complication associated with general anesthesia^[32]^. In this research, it was observed that the incidence of POD in the placebo group stood at 26.11%. Recent studies indicated that 18% of patients who have undergone non-cardiac surgery experience POD^[33]^. In comparison, patients with hyperlipidemia who received a placebo showed a notably higher rate of POD, and this is consistent with previous findings^[7]^. A few studies have been conducted on the preventive therapy of delirium in patients with hyperlipidemia. A recent randomized controlled trial found that the incidence of POD in older patients undergoing joint replacement surgery who received perioperative parecoxib was 17% lower than that in the placebo group^[18]^. This finding aligns with our results, which demonstrate the potential of parecoxib in preventing POD. One major difference is that this study used a more accurate superiority trial and a stricter one-sided test to confirm the POD prevention effect of parecoxib. Furthermore, the single use of parecoxib also reduced the risk of complications associated with NSAIDs^[34]^ and did not elevate the risk of postoperative adverse events in this study. Additionally, we observed that the incidence of ED, which is closely linked to POD development^[35]^, was significantly reduced in the parecoxib group. This indicates that preoperative administration of parecoxib can significantly decrease the incidence of POD among individuals with hyperlipidemia.

POD occurs due to a combination of various factors^[32]^. Our subgroup analysis revealed that higher educational attainment, markedly increased blood lipid level, the presence of carotid stenosis, and greater intraoperative blood pressure variability significantly influenced the therapeutic effect of parecoxib. Individuals who have attained higher levels of education tend to have a greater cognitive reserve, which mitigates the disparity in POD incidence between the parecoxib and placebo groups^[36]^. Elevated blood lipids^[37]^, carotid stenosis^[38]^, and increased intraoperative blood pressure variability^[39]^ can aggravate cerebral ischemia-reperfusion injury, partially offsetting the preventive effect of parecoxib on POD^[40]^. Notably, patients with higher preoperative levels of inflammatory markers (PTGS2 levels, white blood cell counts, and neutrophil counts) showed pronounced cognitive improvement with parecoxib, despite a non-significant interaction. Studies have indicated that higher preoperative levels of inflammation, such as interleukin (IL)-6 levels, are correlated with an increase in POD occurrence^[41]^. This indicates that in patients with hyperlipidemia, the preventive effect of parecoxib on POD may be related to inflammation.

Following various types of stress and trauma during the perioperative period, both peripheral and central immune systems are activated, leading to a significant increase in inflammation throughout the body^[12]^. Subsequent analyses revealed that parecoxib significantly reduced the blood PTGS2 levels, white blood cell counts, and neutrophil counts on the first postoperative day and decreased the pain scores. The perioperative administration of parecoxib has been shown to reduce the level of inflammatory biomarkers, such as systemicIL-6, and tumor necrosis factor-α (TNF-α) in the circulatory system and decrease the VAS pain scores^[18]^. In contrast to these previous studies, our research selected PTGS2, lymphocyte counts, white blood cell counts, and neutrophil counts as indicators of inflammation. Neutrophils and white blood cells are among the most common clinical indicators of inflammation and have been found to play roles in immune infiltration and inflammatory factor release in central nervous system diseases^[42]^. PTGS2, the rate-limiting enzyme for prostaglandin synthesis^[43]^, regulates prostaglandin, TNF-α, and IL-1β levels, triggering neuroinflammation^[16]^. Therefore, parecoxib’s inhibition of postoperative inflammatory cell counts and PTGS2 upregulation, observed in this study, manifests its important anti-inflammatory effects.

Mediation analysis revealed that postoperative PTGS2 levels, white blood cell counts, and pain scores played a mediating role in the relationship between parecoxib and the incidence of POD. Animal experiments have shown that the upregulation of PTGS2-induced inflammation can promote the progression of Alzheimer’s disease^[16]^, and inhibiting the upregulation of PTGS2 can improve postoperative cognition^[15]^. Clinical observational studies have concluded that PTGS2 inhibition^[44]^ and pain^[45]^ are closely correlated with the development of POD. Our findings imply that parecoxib may potentially decrease the risk of experiencing POD by lowering postoperative PTGS2 levels and alleviating postoperative pain. This finding underscores the critical role that adequate postoperative pain management plays in the patient’s prognosis. To understand the dynamic changes in the POD risk associated with inflammation, we conducted an RCS analysis. We found that parecoxib could offset the POD risk of PTGS2 and leukocytes, changing the POD risk of neutrophils from a linear increase to a parabolic shape, suggesting that as inflammation levels increase, the preventive and therapeutic effects of parecoxib on POD are more significant.

The limitations of this study are as follows: First, the postoperative adverse events recorded in this study were primarily pulmonary infections, lower limb deep vein thrombosis, gastric mucosal lesions, and nausea with vomiting. While the incidence of these events should not be overlooked, they may be associated with the major gastrointestinal surgery performed on the patients. Second in addition to safety-related parameters, the economic implications of parecoxib, including its effects on patient hospital costs and length of hospital stay, have not been thoroughly investigated in a systematic manner. Further investigation is required to elucidate the specific and comprehensive impact of parecoxib in order to facilitate its broader application. Third, as POD is a central nervous system disease, cerebrospinal fluid samples might be more appropriate than blood samples for assessing inflammation. Finally, the assessment of POD was only conducted once a day. Despite conducting a thorough medical record review, it is possible that some delirium symptoms or changes may have been overlooked. Future studies should include a multicenter design and more diverse data samples to increase the representativeness of the study population, monitor participants over a longer period, and explore more variables and preventive measures.

## Conclusion

Parecoxib may help in reducing the hyperlipidemia-related POD incidence. The effective anti-inflammatory activity of PTGS2 inhibition by parecoxib and postoperative pain control may be important mechanisms for preventing POD. This study provides robust clinical evidence for parecoxib as a prophylactic treatment for POD in patients with hyperlipidemia.

## Data Availability

The original and analyzed data for the study will be made available upon publication of the article and can be obtained by emailing the corresponding author. The data may only be used for relevant scientific research and must not be used for illegal or commercial purposes.

## Research registration unique identifying number (UIN)

The study was registered in the Chinese clinical trial registry (ChiCTR2300073880) on 24 July 2023. The principal investigator is Duan Xiaoxia.
